# Framing asynchronous interprofessional education: a qualitative study on medical, physiotherapy and nursing students

**DOI:** 10.5116/ijme.6531.02ac

**Published:** 2023-11-06

**Authors:** Matthew Grace, Arden Azim, Sarah Blissett, Amy Keuhl, Sarah Wojkowski, Matthew Sibbald

**Affiliations:** 1 Faculty of Health Sciences, McMaster University, Canada; 2 Department of Medicine, Western University, Canada; 3 School of Rehabilitation Sciences, McMaster University, Canada

**Keywords:** Interprofessional Education (IPE), virtual, asynchronous, module, future collaboration

## Abstract

**Objectives:**

To explore how virtual, asynchronous modules can be used in interprofessional health education curricula and to identify any advantages and shortcomings of asynchronous interprofessional education.

**Methods:**

A sample of 27 health professional students who attended in-person interprofessional education workshops at the McMaster Centre for Simulation-Based Learning from 2019-2020 were recruited through email discourse. Participants were asked to complete an asynchronous interprofessional education module and take part in a semi-structured interview that was recorded and transcribed verbatim. Techniques of direct content analysis were used to analyze the qualitative data from recorded transcripts.

**Results:**

The following emergent themes from participants’ responses were identified: 1) the modules, as well as the features interspersed throughout, taught strategies for conflict resolution and interprofessional communication, 2) the modules have utility in preparing students for future interprofessional learning, 3) the convenience of virtual asynchronous modules introduces a sense of learner safety, and 4) a sense of isolation and fatigue was identified as a consequence of the lack of face-to-face interaction in these modules.

**Conclusion:**

Asynchronous interprofessional education modules may be best suited to prepare students for future interprofessional learning in a synchronous setting. Asynchronous modules effectively provide an introduction to interprofessional objectives such as conflict resolution and role clarification, yet the competency of team functioning is more difficult to achieve in an asynchronous environment. Future studies may focus on establishing a sequence of completing asynchronous modules for ideal development of interprofessional competencies in health professions learners.

## Introduction

Interprofessional education (IPE) represents an important step in advancing health professional education, and is often seen as a key mechanism to improve the overall quality of healthcare.^1^^,^^2^ Due to the disruption to traditional models of interprofessional learning caused by the COVID-19 pandemic, in-person IPE modalities were, and continue to be, shifted to virtual, interactive formats.^3^^,^^4^ Virtual, asynchronous forms of education provide a solution to long-standing logistical and resource barriers to IPE, including the need for dedicated physical spaces and accommodating learners’ schedules.^5^ In a meta-analysis looking at internet-based learning among health professions students, Cook and colleagues found that asynchronous curricula improved educational outcomes with respect to instructional design, study design, and study quality for learners.^6^ What is unknown, however, is whether virtual modules can accomplish the goal of interprofessional learning - learning “from, with and about” other professionals to achieve effective collaboration and improved health outcomes.^7^^,^^8^ In response to the need for better theoretical understandings of asynchronous learning, the objective of this paper is to describe how virtual modules may be used to build a novel theoretical model of asynchronous IPE.

Currently, there are significant challenges in implementing virtual IPE. One considerable challenge comes in the lack of frameworks to guide how virtual IPE may be structured.^9^ Corson and colleagues note there are few relevant frameworks that may inform how interprofessional learning may happen in a virtual setting; existing theoretical frameworks for IPE are based on in-person learning.^10^^-^^12^ Virtual, asynchronous modules add convenience to learners' experiences as students may access educational material in their own preferred environments and at times that work with their schedules.^12^^,^^13^ Consequently, defining a framework that clarifies the implementation of virtual asynchronous IPE curricula would be a significant step towards more effective and convenient education for learners.

Several existing frameworks pertaining to the development of health professional curricula are relevant to informing theoretical models of virtual IPE, including the IPE framework outlined by the Canadian Interprofessional Health Collaborative (CIHC), Bloom’s Taxonomy, and Mayer’s theory of multimedia learning. The IPE framework outlined by the CIHC describes core tenets in establishing interprofessional collaboration. These include competencies such as role clarification, conflict resolution, interprofessional communication, team functioning, collaborative leadership and community/family/patient-centered care.^8^ This framework provides pre-defined competencies to analyze as learners complete virtual asynchronous IPE modules. Moreover, Bloom’s taxonomy presents a hierarchy of various learning objectives that differentiate between varying levels of cognitive ability, and by extension, deeper levels of knowledge transference and application of skills to wider contexts.^14^^,^^15^ In using this framework to assess the efficacy of asynchronous modules, this study will categorize the degree of learning for health professional students after completing virtual lessons.  Further, Mayer’s cognitive theory of multimedia learning posits that learning may be enhanced by the conjunctive use of words and pictures, and the appropriate usage of virtual modalities may improve medical instruction and increase engagement among learners.^16^^-^^18^ These concepts will be considered as participants share their thoughts on design elements of these virtual modules.

Through the use of qualitative data collected from McMaster University health professions students, this study effectively builds upon the evidence-based principles of multimedia design and Bloom’s taxonomy to inform suggestions for the development of asynchronous virtual IPE curricula. We conducted a qualitative study of students engaging in IPE modules at McMaster University to investigate the efficacy of asynchronous virtual IPE curricula. Qualitative data, as opposed to strict quantitative analysis, enable this study to highlight insights and feelings directly from a representative group of participants that would help inform the development of new asynchronous IPE modalities. Through evaluating discussions with students based on their ability to develop/utilize the competencies outlined by the CIHC, this study aims to identify gaps, assumptions and barriers that may manifest in interprofessional learning in a virtual asynchronous setting.

## Methods

### Study Design

This study of health professional learners at McMaster University sought to use qualitative methods to explore how interprofessional learning may occur through the use of virtual, asynchronous modules. This study followed a qualitative content analysis approach in the development of the study’s methodologies. A content analysis approach in the context of this study involved the usage of previously established theories and frameworks (such as Bloom’s Taxonomy and the CIHC competencies) to inform conclusions that supported the application of these frameworks to a virtual, asynchronous environment. The content analysis methods in this study involved the subjective interpretation of interview transcripts, comparison of categories and codes, and the identification of connections between existing theories and study data.^19^ Moreover, the study followed a deductive approach in order to test existing theories of interprofessional learning in a new context.^20^^,^^21^

### Study Participants

The term “participant” refers only to those recruited in this study, while the phrase “learner” can denote those students in this study as well as the general body of students that the study findings apply to. Participants were recruited from health professions students who attended in-person IPE workshops at the McMaster Centre for Simulation-Based Learning (CSBL) from 2019-2020. These participants had previously attended in-person IPE workshops at the McMaster CSBL, and the list of potential participants was comprised entirely of learners that had provided verbal permission to be contacted about new IPE initiatives. Potential participants were identified based on the following eligibility criteria: (i) a current student pursuing a health professions degree (nursing, medicine, physician assistant, etc.), and (ii) previous experience with in-person IPE at McMaster. Ninety-three eligible learners were invited to participate in this study through email and were given the opportunity to address any questions about the study. Additionally, a convenience sample was used for the recruitment of one physiotherapy student who completed the modules as part of their placement activities. During enrollment, participants were informed of potential privacy concerns, such as the recording of interview discussions. Participants were told that their data would remain confidential through anonymization. Twenty-seven learners from three different professional programs (9 nursing students, 13 medical students and 5 physiotherapy students) consented to participate in this study. This study was granted ethical exemption from the Hamilton Integrated Research Ethics Board (HiREB) in December 2021 as the study was classified as quality improvement.

### Data Collection

Consent was obtained via email, and a meeting time was arranged between the interviewer (MG) and each participant. During the video calls that took place on the licensed video conferencing software Zoom,^22^ participants were asked to complete one of five virtual asynchronous modules developed by McMaster CSBL staff and faculty in 2021 and 2022 (Appendix A). During module completion, microphones and cameras were muted/turned off to simulate an asynchronous environment. Following completion of the module, participants would engage in a semi-structured interview with MG. An interview guide was developed based on previous IPE studies and an extensive literature review.^9^ The guide included open-ended questions with follow-up prompts to explore participants’ experiences with virtual learning, IPE, and participants’ perceptions of how the modules facilitated learning of the interprofessional competencies outlined by the CIHC. Discussions also centered around suggestions for implementing these modules into health professions’ curricula.  During these discussions, the CIHC competencies were provided for participants to reference in their responses. The semi-structured interviews concluded once each participant expressed that they had said everything they wanted to share. Audio from each of the interviews were recorded and transcribed clean verbatim.  Interviews were conducted until the research team agreed thematic saturation was achieved, whereby no new themes were emerging between interviewees.^23^ Biweekly meetings with the research team were held to discuss the sufficiency of the data collected at regular intervals during the data collection period. The authors agreed that data saturation had been achieved considering that later participants provided similar insights to previous participants. The research team felt confident that the data collected reflected a variety of perspectives and held several themes to draw conclusions from. The collection of these interview responses occurred between October 2021 and March 2022.

### Rigor and Trustworthiness

The research team represents a diversity of professional perspectives. MG is an undergraduate student with experience in university-level virtual learning. AA, MS, SB and SW are educators with experience in interprofessional education. AA has training with nursing; SW within rehabilitation sciences; AA, SB and MS within medicine. Each team member holds their own experience with respect to IPE, virtual learning, and curriculum development for health professions. The research team holds the same understanding about the function of IPE, and what constitutes as learning “with, from and about different professions” in the context of IPE. All members of the research team were provided context with respect to the purpose of the study before engaging with the data that were collected. Each member was given the opportunity to explore the transcripts and make comments individually prior to consulting with the rest of the research team. To promote reflexivity, monthly meetings were held where the research team would discuss findings from the interview transcripts.^24^ Meeting notes were recorded to capture different insights presented by the team.

### Analysis

The interview transcripts were analysed through applying techniques of direct content analysis.^25^ This process included planning, contextualisation, categorization, and compilation.^19^ In planning, transcripts were coded independently by each member of the research team, and following discussions about recurrent themes, the research team generated a list of codes to inform the analysis of the transcripts, reflecting an open coding process.^26^ During the contextualisation and categorization stages of analysis, the transcripts were re-read with the intention of assigning meaning to the interview responses and sorting certain themes together, reflecting an axial coding process.^27^ Initially, the codes were informed by different frameworks, including the CIHC IPE competencies, Bloom’s Taxonomy, and feedback/suggestions regarding the modules and their features. Preparation for Future Learning (PFL) was added as an additional theoretical paradigm for analysis following insights pertaining to continued problem solving for health professions learners that came from analyzing the codes.^28^ Finally, during the compilation phase of data analysis, the research team considered how the codes related to existing literature about IPE, and notable conclusions from discussions with the research team were captured. All discrepancies between coders were resolved through discussion, and consensus was achieved.^29^^,^^30^ Codes were captured and analyzed using the analysis software Dedoose.^31^ Data analysis concluded once the research team agreed that the codes had achieved conceptual depth, and the findings were externally applicable to the health professions education and academic communities.^25^^,^^32^

## Results

The participants represented a variety of health professions student experiences with participants coming from different levels of education. Table 1 describes the participant demographics as well as the module designations for each participant. Only demographics relevant to the study were collected. Four recurrent themes were identified from discussions with the participants. 

### Theme 1: Helpful virtual module features

When asked about aspects of the modules that facilitated learning about interprofessional competencies, many students replied that there were unique features in the virtual modules that they appreciated. Specifically, the videos, reflection prompts, and checklists were features commonly identified as standouts to participants (Appendix B).

“The videos were good and I like the comparisons, so it was nice. I like the very clear explanation of the acronyms, as well as ways to improve your way to relay that information.” – Participant 20, third-year medical student

**Table 1:** Demographics and Module Designations for Participants

Program	Year Level	Number of Participants	Module 1: Workplaces	Module 2: Feedback	Module 3: Handover	Module 4: Debriefing	Module 5: Virtual Care
Medicine		13					
	2^nd^ year	10	3	1	2	1	3
	3^rd^ year*	2	0	0	1	1	0
	PGY 2	1	0	0	0	0	1
Nursing		9					
	3^rd^ year	4	1	2	0	1	0
	4^th^ year	5	1	2	1	1	0
Physiotherapy		5**					
	1^st^ year	5	1	1	1	1	1
Total		27	6	6	5	5	5

* McMaster University has a three-year medical school therefore third-year medical students are in their final year. ** The modules counted towards the physiotherapy learner’s placement activities, so a convenience sample was used to recruit this participant. This participant completed all five modules.

“I like the fact that there were opportunities throughout the entire module to type up your answers.” – Participant 13, fourth-year nursing student

Several participants identified that the videos in the modules were helpful in visualizing certain scenarios, allowing learners to internalize different CIHC IPE competencies, such as conflict resolution and role clarification.

“I liked the conflict resolution part as well, and those two different videos and how they showed that in order to just resolve a conflict, you need to own up to your mistake and work together in order to solve it.” – Participant 14, third-year nursing student

Participants often shared that the videos, in conjunction with the assessment questions and reflection prompts, taught learners effectively about conflict resolution concepts. The progression of the concepts taught in each module was something participants expressed as being helpful in learning about conflict resolution.

“The interactive part of it, filling in the questions and stuff. And... I liked the video. I thought those were helpful in terms of just seeing the different examples of receiving feedback and what the effective response was and how receiving feedback could be made more productive.” – Participant 11, fourth-year nursing student

### Theme 2: Preparation for Future Learning

A salient theme from the interviews was the idea that these asynchronous modules could be used to prepare students for learning in future situations. Several participants provided suggestions for how the modules can be implemented into existing IPE curricula. A common suggested use for the modules was to provide context for learners for future interprofessional interactions.

“I think, in general, it definitely does help with the introduction to the concept of interprofessional collaboration” – Participant 22, second-year medical student

“So more so focusing on the future aspect, maybe asking people to go through the steps by themselves at the end... Just so they have a better understanding of what to do in the future.” – Participant 19, fourth-year nursing student

Despite the absence of interprofessional dialogue during the completion of these modules, participants found that the modules addressed concepts relevant to pre-clinical learners. Some participants stated that the modules were helpful in reminding them of IPE concepts, while others shared that the modules would have enhanced their learning experience if they were completed prior to formal instruction.

“…the module is a good introduction, and I feel like this actually would have been nice before start of the lectures to have something like this” – Participant 9, second-year medical student

Consequently, asynchronous modules can function to introduce exploratory activities and encourage discovery first before formal instruction as an effective way to promote flexible learning in the future.

### Theme 3: Learner safety

Participants described the asynchronous nature of these modules as beneficial in terms of instilling a sense of psychological safety.

“You had that little break to kind of collect your thoughts again and kind of restart.” – Participant 5, fourth-year nursing student

“Honestly, I think it's quite better. It's at your own pace rather than listening to someone lecture, and then you do your own thing.” – Participant 6, second-year medical student

Moreover, participants felt that it was difficult to ask questions during in-person clinical situations, but after completing these modules, some participants felt that these virtual lessons served as reminders that let learners know it was safe to ask questions in the future.

“As a student, sometimes it's hard to initiate debriefing, which I think was recognized in the module as well, that students can actually do that. We're allowed to do that.” – Participant 13, fourth-year nursing student

The modules were perceived to help participants learn about effective interprofessional interactions, and several participants stated that they would be more comfortable handling conflict or speaking with other professions. Beyond creating a space to remind students about question-asking, some participants identified that the asynchronous nature of these modules was convenient for students to take their time and internalize IPE concepts at their own pace.

“I think they facilitate learning because they allow you to go at your own pace… especially if you need that time to process any information that you're given. And as well, it's also easy to access so that you can go back to it if you have any questions, or if you forgot something.” – Participant 21, third-year nursing student

Accordingly, many participants explained that they were able to internalize IPE concepts because the presentation of the information was suited to different learning styles. Additionally, some participants shared that the asynchronous timing of these modules added an extra layer of convenience; participants found this to be a beneficial feature of the modules compared to in-person instruction.

“An online module like this makes it a lot easier to fit it into everyone's busy schedules and to do things when you're able to and on your own time.” – Participant 22, second-year medical student

### Theme 4: Zoom and module fatigue

Module fatigue and Zoom fatigue were common shortcomings of virtual learning identified by participants. In the context of the recent pervasive implementation of virtual curricula due to the COVID-19 pandemic, participants expressed a sense of exhaustion from completing these modules, and commonly shared that this may diminish the quality of one’s learning.

“...especially if there's multiple modules in a row, it can be really exhausting to do a whole bunch of them.” – Participant 18, second-year medical student

Also, some participants felt that it might be hard to sustain attention for the duration of the lesson. Participants often compared the modules to in-person instruction, and the lack of accountability was a considerable factor in completing virtual modules.

 “It's very easy to kind of not pay attention like in-person, of course, and especially if you're getting called on or you know that you're gonna have to re-enact this with other people you're working with. You're definitely gonna pay a lot more attention.” – Participant 16, recently-graduated nursing student

Additionally, participants shared that the lack of actual face-to-face interaction was discouraging, and, as a result, certain IPE competencies may be difficult to learn about in an asynchronous setting, such as team functioning and collaborative leadership.

“Not having that face-to-face interaction, as well as not hearing about people's stories, which I think is one of the most pertinent ways to learn, is just hearing people's experiences. I think those are some of the challenges in terms of understanding other experiences.” – Participant 20, third-year medical student

“Maybe like the collaborative leadership part. I don't think the module had any specific things for collaborating to form like a treatment plan or something like that.” – Participant 7, second-year medical student

Participants shared that the lack of conversation with other professionals also made it difficult to achieve the goals of these IPE competencies. Moreover, some participants felt that examples of team functioning and collaborative leadership needed to be more explicit in order to effectively teach them.

“…collaborative leadership, I didn't really feel like the module really covered that part.” – Participant 14, third-year nursing student

## Discussion

### Role Clarification

Study findings indicate that core CIHC interprofessional competencies, such as role clarification, can be achieved from conducting IPE in a virtual asynchronous manner. Role clarification is an essential concept for learners to understand as they transition into work with health care teams.^8^^,^^33^ Health professionals must understand their roles and the roles of other professionals in the health care team to effectively collaborate to achieve patient goals.^8^^,^^34^ Effective role clarification leads to improved coordination of care and the appropriate utilization of each professional’s expertise.^35^ Conversely, role ambiguity may result in interprofessional tension and gaps or duplication in the delivery of health care services.^36^^-^^38^The modules assessed in this study were effective in teaching role clarification as participants felt the videos and the reflection prompts in the modules highlighted understanding one’s role to a significant degree. The videos provided adequate examples of how each of the different professions may function in a health care setting, exemplifying effective role clarification. Also, the reflection prompts interspersed throughout the modules gives learners an opportunity to consider what their role in a given situation might look like.

### Conflict Resolution

In addition to role clarification, study findings indicate that conflict resolution is another core CIHC interprofessional competency that can be achieved from conducting IPE in a virtual asynchronous manner. Conflict resolution refers to a professional’s ability to engage oneself in discussion with other professionals and patients/families/clients when dealing with disagreement or conflict.^8^^,^^34^^,^^36^^-^^39^ With effective conflict resolution, positive team dynamics can be fostered, and shared problem solving and acceptance of change can be encouraged.^40^ Teaching IPE concepts directly through text and imagery, followed by a video example and questions for reflection, may be an effective way for asynchronous virtual modules to help learners develop an understanding about concepts such as conflict resolution. From the perspective of Bloom’s Taxonomy, the modules provide learners with an opportunity to understand and analyze interprofessional healthcare scenarios while also visualising how these concepts can be applied to their practice in the future.^14^^,^^15^ Participants’ comments reinforced that the modules effectively achieved learning outcomes associated with role clarification and conflict resolution; this aligns with current studies assessing virtual interprofessional education.^9^^,^^41^ The opportunity to conduct these modules asynchronously allows for the addition of beneficial learning features, such as videos, reflection prompts, and checklists.

### Preparation for Future Learning

Preparation for Future Learning (PFL) involves using current resources to adapt in learning new information and to support learning during practice.^28^ Given the asynchronous nature of the virtual modules, learners are encouraged to explore IPE concepts by taking initiative in their own learning. The consideration of preparing students for future learning in curriculum design may lead to effective problem solving in practice for health professionals.^21^^,^^28^ Without direct instruction, as in asynchronous modules, learners are encouraged to come up with their own solutions to problems, effectively developing PFL-related behaviours. ^28^^,^^42^^-^^44^These behaviours may include seeking feedback, teaching/learning from colleagues, and asking pertinent questions to other professionals.^45^ As learners are encouraged to develop their own questions, generate their own ideas, and compare different cases through completing the modules, students can be better equipped to learn about interprofessional competencies.^46^ Asynchronous education may effectively prepare health professional students for collaborating in practice in the future, and the modules are particularly effective at achieving the goals of individual exploration and generation. In designing asynchronous IPE curricula, there should be a balance between exploration and direct instruction to achieve the goals of PFL.^28^ While direct instruction may initially appear to be an efficient instructional methodology, it introduces the danger of overly narrowing a learner’s focus.^47^ Informed by responses shared by several participants, it is suggested the asynchronous modules be accompanied by synchronous discussion to achieve maximum educational benefit. The asynchronous modules can be used to prepare students for learning about these competencies in the future where synchronous discussion may take place. When considering the framework of IPE in terms of future learning, the concept of Preparation for Future Collaboration (PFC) can be used to describe how the modules encourage the development of IPE competencies for future use.45 Ultimately, with the identified need in healthcare for experiential learning opportunities set in the clinical environment,^48^ learning about team functioning and collaborative leadership from asynchronous IPE modules could be enhanced when followed by synchronous discussion.

### Learner Safety

Feelings of safety in a virtual learning environment are often associated with flexibility, freedom to learn at one’s own pace, and easily accessible learning materials.49 The asynchronous modules, with questions and prompts provided throughout the modules, give students an opportunity to become more self-reflective. This offers learners the chance to confront debatable ideas in a more reflective and objective manner.^50^^,^^51^ Participants’ comments about becoming more confident in asking questions relates directly to the concept of self-advocacy for learners. The data from this study suggest multiple participants felt empowered to have interprofessional conversations in the future as a result of the concepts taught in the modules. Furthermore, participants identified beneficial module features that allowed them to learn about IPE concepts, indicating that the layout of the modules is effective and adheres to Mayer’s principles of multimedia learning. Participants felt the modules were effective because learning objectives were clearly outlined before each chapter, text was presented in succinct points accompanied by images and acronyms, and supplementary handouts were provided for participants to download after the module was completed.^16^^-^^18^^,^^52^^,^^53^ These features help overcome the cognitive load of processing several problems at once,54 which is reduced in an asynchronous learning space compared to in-person instruction. These factors contribute to learner safety, and this process of guided discovery for learners allows students to reflectively explore IPE curricula. Guided discovery is most effective when followed by direct instruction at a later time, offering learners an opportunity to consider learning “from, with and about other health professionals” in the future.^28^^,^^55^

### Module and Zoom Fatigue: Collaboration

Team functioning and collaborative leadership, competencies outlined by the CIHC, both deal with the necessity of sharing perspectives and responsibilities with other professionals and patients in order to achieve greater health outcomes.^8^^,^^56^^,^^57^ As reported by participants, these skills are more difficult to achieve in an asynchronous environment since pre-programmed modules are unable to represent the contextual issues that are associated with face-to-face experiences. Moreover, while asynchronous modules can be completed at one’s own convenience, the exhaustion from virtual learning may diminish learners’ abilities to internalize IPE concepts. When implementing virtual asynchronous modules into health professional students’ curricula, it is suggested that learners are reminded to take periodic breaks. Taking breaks may lower mental stress and screen fatigue, allowing learners to re-engage with the material with a refreshed sense of clarity.^58^^,^^59^ Asynchronous learning modalities enable learners to take breaks at their own volition, leading to improved learning outcomes and enhanced understanding of IPE competencies.

### Limitations

This study has important limitations. Participants in the study were asked to complete a module at once in its entirety for the purpose of conducting interviews afterwards. In reality, learners would have the option of completing the modules on their own agendas, can pause and go back to concepts as they see necessary, and can take breaks before completing successive modules. Consequently, these considerations may alleviate the feelings associated with module fatigue identified by the participants in the study. Further, the sample size was relatively small and only represented three health professions; however, it was decided that the enrollment of additional participants was not necessary considering the research team’s agreement that thematic saturation had been achieved.^23^ Despite these limitations, this study is strengthened by having a sample that represents professional learners at different stages of their training, and by assessing a variety of virtual modules that deal with several salient topics in health professions education. As a result, this study makes important contributions to the literature on the implementation of asynchronous virtual modules into health professional curricula.

## Conclusion

Virtual asynchronous modules introduce several benefits for health professions students as they learn about interprofessional competencies. Beyond the convenience of being able to integrate the modules into learners’ busy schedules, reflective module features enable students to understand their roles to a greater degree, and to consider how they might advocate for their own learning in the future. Role clarification and conflict resolution are competencies that can be effectively taught in a virtual asynchronous space, whereas the goals of team functioning and collaborative leadership are difficult for asynchronous curricula to achieve. At a systems level, these findings suggest interprofessional learning can happen in a virtual, asynchronous setting, and virtual modules are an important tool in medical education that is effective in preparing students for future learning. Further, while there is no inherent order for learning IPE competencies, certain modules may teach particular competencies to different degrees, and future studies may find it worthwhile to explore how this format of IPE delivery might be sequenced in a health profession learner’s development.

### Acknowledgements

We would like to thank the staff at the McMaster CSBL for providing us with modules to use in this study.

### Conflict of Interest

The authors declare that they have no conflict of interest.

## Supplementary material

Supplementary file 1 Appendix A. Summary of content in each of the modules presented to participants

Supplementary file 2 Appendix B. Supporting excerpts for discussing CIHC Competencies
