# Supplementary material for: Framing asynchronous interprofessional education: a qualitative study on medical, physiotherapy and nursing students
Source: Int J Med Educ. 2023 Nov 6;14:155–67. doi: 10.5116/ijme.6531.02ac (PMC10693952; doi:10.5116/ijme.6531.02ac)
Supplement: Supplementary file 1 — Appendix A. Summary of content in each of the modules presented to participants [file ijme-14-155-S1.pdf]

Appendix A

Summary of content in each of the modules presented to participants

Module title	Learning Objectives	Video Scenarios	Assessment Checkpoints	Reflection Prompts	Sorting Activity	Checklist Activity
MODULE 1: TRANSITION TO WORKPLACE LEARNING	• Recognize your role as professionals-in-training in the workplace<br>• Describe roles and role limitations of other professionals in the interprofessional healthcare team<br>• Resolve patient-care related conflict in the workplace between learners in two professions<br>• Develop a framework for collaborative decision-making in the clinical workplace	Found in this module<br><br>Example: *Articulating your Role as a Professional-in-Training*	Found in this module<br><br>Example: *What should be included when articulating your role in the workplace?*	Found in this module<br><br>Example: *Why does role clarity matter? Discuss this scenario.*	Found in this module<br><br>Example: *What is Workplace Learning? Drag-and-drop activity*	NOT found in this module
MODULE 2: FEEDBACK LITERACY	• Recognize the value and role of feedback from interprofessional sources<br>• Understand ways to manage the impact of affective response (emotions, feelings and attitudes) in response to feedback<br>• Develop an approach to processing and reciprocating interprofessional feedback<br>• Develop and plant to respond and act on interprofessional feedback	Found in this module<br><br>Example: *What is feedback?*	Found in this module<br><br>Example: *Select the most appropriate strategy to manage negative feedback.*	Found in this module<br><br>Example: *Write down a few notes about a time you received feedback.*	Found in this module<br><br>Example: *Rank the following sources based on how valuable you perceive their feedback to be.*	Found in this module<br><br>Example: *Learner Toolkit for Feedback Literacy – Steps Before Feedback*
MODULE 3: HANDOVER OF CARE	• Understand the principles and importance of safe and effective interprofessional handover<br>• Integrate structured communication tools into patient handover in their own practice setting<br>• Identify strategies to communicate urgency and raise concerns in the interprofessional clinical setting	Found in this module<br><br>Example: *Challenges for interprofessional communication*	Found in this module<br><br>Example: *Which of the following are facilitators of effective handover?*	Found in this module<br><br>Example: *Write down an example of handover that you have observed.*	NOT found in this module	Found in this module<br><br>Example: *Learner Toolkit for Handover – Applying SBAR and PACE*
MODULE 4: DEBRIEFING WORKPLACE EVENTS	• Articulate the role and importance of debriefing workplace events<br>• Explain different types of workplace debriefing, and how they can be integrated into the clinical workplace<br>• Apply an organized framework for debriefing clinical events	Found in this module	Found in this module<br><br>Example: *Which strategies can help you initiate*	Found in this module<br><br>Example: *Write down a few notes about a time where you felt*	NOT found in this module	Found in this module<br><br>Example: *Learner Toolkit – Strategies*

	• Recognize factors required to establish and maintain a psychologically sage environment for debriefing workplace events<br>• Define performance gaps and strategies for identifying them<br>• Develop an action plan for integrating workplace event debriefing into their clinical training	Example: *Setting the Stage for Debriefing – Video example*	*debriefing as a learner? Select all that apply.*	*that you participated in or witnessed a debrief.*		*to Integrate Debriefing*
MODULE 5: VIRTUAL CARE	• Understand approaches to integrating virtual care technologies into an interprofessional patient care plan<br>• Demonstrate an understanding of the professional role in virtual care, and an approach to translating professional competencies to virtual patient care<br>• Develop an approach to interprofessional and patient/client-centered communication in the virtual space<br>• Plan an approach to a virtual care patient visit	Found in this module<br><br>Example: *Telehealth Etiquette - Example scenario*	Found in this module<br><br>Example: *Select which patient might be most appropriate for a virtual care visit.*	Found in this module<br><br>Example: *Write down ideas related to benefits, challenges, and ethical dilemmas in virtual care.*	NOT found in this module	Found in this module<br><br>Example: *Learner Checklist for Virtual Care – Before the Visit*
