# Supplementary material for: Framing asynchronous interprofessional education: a qualitative study on medical, physiotherapy and nursing students
Source: Int J Med Educ. 2023 Nov 6;14:155–67. doi: 10.5116/ijme.6531.02ac (PMC10693952; doi:10.5116/ijme.6531.02ac)
Supplement: Supplementary file 2 — Appendix B. Supporting excerpts for discussing CIHC Competencies [file ijme-14-155-S2.pdf]

Appendix B

Supporting excerpts for discussing CIHC Competencies

Table 1. CIHC Competencies Achieved by Module Features

**Feature**	**CIHC Competency**	**Notable Excerpt(s)**
**Videos**<br><br>*The videos included in the modules were fictional scenarios recreated by McMaster CSBL staff that aimed to replicate and visualize healthcare situations.*	Role Clarification,<br><br>Interprofessional<br><br>Communication	*“I think just having that role clarification and having, perhaps, point of views from different professions mentioned in the videos… was helpful.” - Participant 14*<br><br>*“I liked the video. I thought those were helpful in terms of just seeing the different examples of receiving feedback and what the effective response was and how receiving feedback could be made more productive.” - Participant 11*
**Reflection Prompts**<br><br>*Theses prompts were questions asked to participants, and were often hypothetical in nature (e.g. “what would you do in this situation?”). Text boxes were provided along with these prompts so that learners could record their responses.*	Conflict Resolution, Role Clarification,<br><br>Interprofessional<br><br>Communication	*“I learned that it's important to communicate with each other, whether it be preceptor to student, students' ability to initiate a debrief. I learned that it's really important to communicate, to recognize what your roles are, to recognize how you fit in into this whole teamwork or team function kind of approach, really.” - Participant 13*<br><br>*“Clarifying roles is an important factor and then being able to resolve conflict without actually having conflict by communicating properly between practitioners, I find was effective. And then just reflecting, really understanding, the clarity of other people's roles as well as your own is important, so I would say those were important parts.” - Participant 10*
**Checklists**<br><br>*The checklists are usually found at the end of each of module subsection, and it provided learners with a clickable box beside each learning goal so that participants could track their progress throughout the module.*	Family/Patient/<br><br>Community-<br><br>Centred Care	*“In terms of handover, I absolutely believe that's between healthcare professionals, but also though with families, checklists were maybe a way for me to see how to provide healthcare information to the families in a comprehensive way.” - Participant 20*<br><br>*“I really like the flow and the checklist, I think anything that's really practical always works very well 'cause it's a nice easy thing to remember in terms of virtual care.” – Participant 4*
**Text on Slides**<br><br>*Text was often accompanied with graphics, and text was commonly used to directly teach IPE concepts, usually in bullet points or drop-down lists.*	Team Functioning, Role<br><br>Clarification	*“Definitely the content on team functioning as well in terms of... Feedback is essential for team functioning, in my opinion, and even just like getting to know the interprofessional team and respecting all of their opinions and different perspectives, I think that sort of falls under team functioning as well.” - Participant 11*

Table 2. CIHC Competencies Difficult to Achieve by Asynchronous IPE

**Module**	**CIHC Competency**	**Notable Excerpt(s)**
Virtual Care	Collaborative Leadership	*"Yeah… collaborative leadership, I didn't really feel like the module really covered that part." - Participant 14*
Handover of Care	Team Functioning	*"So the challenges is that it's very different from real life both with the verbal aspect of communication, but then the verbal communication aspect, but then also the acuity sometimes of handover or the real life scenario where... It's not just you, it's you plus, it could be a team of, I don't know, five to 10 other health professionals." - Participant 7*<br><br>*"I think one of the main challenges is that it kind of becomes more individualized and you don't get to see what other people are thinking, so a little bit more isolating if it's just the module that you do on your own." - Participant 18*

Table 3. CIHC Competencies Mentioned by Module

**Most Pertinent Module**	**CIHC Competency**	**Number of Mentions**
Transition to Workplace Learning	Role Clarification	28
Handover of Care	Team Functioning	27
Transition to Workplace Learning	Conflict Resolution	16
Feedback Literacy	Collaborative Leadership	12
Handover of Care	Interprofessional Communication	32
Transition to Workplace Learning	Patient/Family/Community-Centered Care	5
